# A Case Study of Adding Proactivity in Indoor Social Robots Using Belief–Desire–Intention (BDI) Model

**DOI:** 10.3390/biomimetics4040074

**Published:** 2019-11-20

**Authors:** Ujjwal K. C., Jacques Chodorowski

**Affiliations:** 1 Discipline of ICT, University of Tasmania, Hobart, Tasmania 7005, Australia; 2 Orange Labs, 2 Avenue Pierre Marzin, 22300 Lannion, France; Jacques.Chodorowski@orange.com

**Keywords:** social robots, proactivity, belief–desire–intention (BDI) model, robot operating system (ROS)

## Abstract

The rise of robots and robotics has proved to be a benefaction to humankind in different aspects. Robotics evolved from a simple button, has seen massive development over the years. Consequently, it has become an integral part of human life as robots are used for a wide range of applications ranging from indoor uses to interplanetary missions. Recently, the use of social robots, in commercial indoor spaces to offer help or social interaction with people, has been quite popular. As such, taking the increasing use of social robots into consideration, many works have been carried out to develop the robots to make them capable of acting like humans. The notion behind this development is the need for robots to offer services without being asked. Social robots should think more like humans and suggest possible and suitable actions by analyzing the environment where they are. Belief–desire–intention (BDI) is one of the most popular models for developing rational agents based on how humans act based on the information derived from an environment. As such, this work defines a foundation architecture to integrate a BDI framework into a social robot to add “act like a human” feature for proactive behaviors. The work validates the proposed architecture by developing a vision-based proactive action using the PROFETA BDI framework in an indoor social robot, *Waldo*, operated by the robot operating system (ROS).

## 1. Introduction

In this section, we first discuss the basic concepts of social robots and proactivity and their relevance in our daily lives. We then converge these concepts to form the primary objective of creating additional value by adding proactivity in robot operating system (ROS) powered indoor social robots by using a belief–desire–intention (BDI) model.

### 1.1. Social Robots

The idea of the robot gives an impression of human-like machines that are primarily created to serve their creators. The way how robots serve the human has a broader scope. The robots can be used in daily household chores to space exploration missions depending upon how they are built and how much intelligence is installed on them. With the evolution of time, the way to define a robot has changed from *“a term for mechanical men that were built to work for assembling products in a factory”* to *“an entity which humans can have social interactions with”*. The inception of social robots was biologically inspired, and social robots were initially used to study the swarms or the behavior of insects [1]. Later, social robots were used for interaction with humans.

According to social scientist Kate Darling, *“A social robot is a physically embodied, autonomous agent that communicates and interacts with humans on an emotional level”* [2]. For this article, it is important to distinguish social robots from inanimate computers and industrial or service robots (not designed to elicit human feelings and mimic social cues). Social robots also follow social behavior patterns, have various “states of mind”, and adapt to what they learn through their interactions. Usually, the social robots are in humanoid or animaloid form to create an emotional connection with the human as forms and shapes of social robots are very important. The social interaction is expected to be similar to verbal communication with visual and tactile perception. Based on the interactions, social robots can be classified into the following categories [3].

1. Socially evocative. These robots rely on human action to generate a particular set of feelings [4].
2. Socially situated. These robots react to the perceptions derived from the social environment in which they are situated. The robots can distinguish the social agents and objects in the environment [1].
3. Sociable. These robots have models for social cognition and proactively engage with the human for some social aims [4].
4. Socially intelligent. These robots try to replicate the social intelligence of humans based on the model of cognition and social competence [5].

The rise of demand for personal care for ageing people and technological advancements have made it possible for social robots to be used extensively for elderly care. Assistive technologies in the form of social robots help the older population to live an independent life in their homes. Social robots can provide a wide range of interactive services like telecare and robot-assisted therapy. Such robots can be used for patient care for older people with mental and cognitive impairments as well [6]. Some of the social robots can monitor the movement, blood pressure, breathing or heart problems and warn a related person in case of any danger or risk [7]. Social robots have been used for a wide range of interactions with children as well. A social companion robot called ’Arash’ was built to provide therapeutic intervention in pediatric hospitals [8]. The social robot was also utilized to assist the children with cancer [8]. Social robots have been recently reported to be used in the education and care of children with developmental disabilities [9]. Proactive robots have been used as tutors and peer learners to deliver education as presented in [10]. As such, social robots are currently used in hospitals, homes, shopping malls, and convention centers to interact with people to either welcome, converse or take care of the people [11]. Social robots have taken challenges of interacting with, assisting, serving and exploring with humans to help humans in different ways. For these applications, proactive behaviors are necessary for social robots.

### 1.2. Proactivity

Social robots are autonomous robots that communicate with humans following a set of social rules defined for them [12]. These robots use three different control architectures to decide the actions required to respond to the environment—deliberative, reactive and hybrid. In deliberative control, robots have thoughtfulness in decision making as there are capacities to relate to the past or future states, beyond the present sensor inputs and stimuli, to take relevant action. Reactive control is similar to ‘stimulus-response’ control mechanism in which the robots respond very quickly to changing and unstructured environments by tightly coupling the sensory inputs and effector outputs. In the hybrid control, one component deals with planning the actions while other deals with immediate reactions that do not usually require learning abilities. The coupling of two different mechanisms in hybrid architecture can be difficult as two control mechanisms have to communicate with each other continuously.

Based on these control architectures, a social robot can interact with humans in two ways. The robots can be asked to do things for humans and in such cases, the robots are reactive. In contrast, robots can automatically help the user without being asked, and in such cases, robots are proactive. The notion of proactivity in social robots can be a useful utility as social robots are primarily meant to interact with the human in a more humanly ways. An example (adopted from [13]) to understand the difference between reactive and proactive behavior in social robots is included in Appendix A.

### 1.3. Robot Operating System (ROS)

ROS is an open-source, C++ based, a general software framework for robot software development which gives operating system functionalities [14]. Those functionalities are hardware abstraction, low-level device control, implementation of commonly-used features, message-passing between processes, and package management. It is based on a graph architecture where each node receives and process several messages from/to sensor, actuators about their state. This operating system works on top of Linux (Ubuntu) and can be used in Windows with some reduced features. ROS creates an ecosystem where different components called nodes are interconnected using a system of message communication between them. ROS is one of the key driving components, that power the commercial indoor robots.

### 1.4. BDI

BDI is one of the major approaches to build a multi-agent system by intelligent programming agents. This model is inspired by human reasoning and based on three entities, namely, beliefs, desire, and intentions [15]. It gives a mechanism for separating the activity of choosing an action from the execution of currently active plans. The agents in a system are defined in terms of these entities. This model also considers the resource bound so that an intention will result after the agent’s reasoning. BDI model expects the agent to act in a dynamic environment such that the agent’s reasoning should take the environment changes into account to make an action. The three entities of a BDI model are explained as follow:

1. Beliefs. Beliefs are the entities that represent the informational state of the agent. Beliefs reflect the knowledge of the robot. Beliefs are stored in belief sets.
2. Desires. Desires are the entities that represent the motivational state of an agent or the goals, objectives or situation which the agent would like to achieve. Desires are what the agent wants to accomplish.
3. Intentions. Intentions are the entities that represent the deliberative state of an agent. Plans are sequences of actions that are taken by an agent to accomplish the goal.

For social robots, the sensor outputs build the belief sets to signify the environment around the robots in terms of different values of different parameters. A particular belief set describes a specific situation in which the robot is located in a specific instant of time. Based on the location, a goal is defined for the robot which can be referred to as desire. A BDI interpreter or engine selects a particular action (intention) from a plan library, which is a collection of intentions, based on the situation.

### 1.5. Rationale

Social robots have become integral and inseparable parts of our life. From the hospitals to convention stores like home, social robots are being extensively used to interact with the human in one way or another. The social robots are now considered as a substitute for human wherever and whenever human is not able to be present for the specific tasks. The robots, therefore, are expected to act more as humans and proactivity can be the feature in such robots that will enable this capability. Many studies have tried to develop proactive behaviors in robots. But when it comes to the commercial indoor social robots powered by ROS, there are no well-defined system architectures and implementations. Given the influence of such robots in a commercial indoor environment, this study tries to explore the possibilities of enhancing proactivity in such commercial indoor social robots. This work defines a clear method to implement different activities of a social robot as proactive behaviors by integrating human reasoning-based BDI framework into the system architecture. The work validates the implementation by developing a vision-based proactive behavior enabled by PROFETA (BDI) framework in an indoor social robot, *Waldo*, operated by ROS. The specific contribution of the study are listed below:

1. A validated modular system architecture, with features such as modularity, flexibility, and rational work distribution, that facilitates different logical blocks to be integrated with robots controlled by ROS for proactive behaviors.
2. A foundation for the development of human-like behaviors in ROS-controlled robots for daily-life applications.

The rest of the paper is organized as follow: Section 2 discusses the related work, while Section 3 explains the case study set up along with the system overview. Section 4 discusses the results, while Section 5 concludes the paper.

## 2. Related Works

In this section, we describe the background of different BDI models and different frameworks built to integrate behavior models in robots. Furthermore, we present different works where researchers have tried to incorporate such models in robots for various daily life applications.

### 2.1. BDI Model

Whenever the design of the cognition model for software agents comes into play, the BDI model is one of the most popular architectural choices. BDI models provide an explicit and declarative representation of informational attitudes, motivational attitudes, and deliberative commitments. Myers et al. [16] divided the BDI models into two broad categories of B-DOING and Delegative models. In the B-DOING model, motivational attitudes are highly adapted, and desires correspond to what the agent wishes. Furthermore, obligations corresponded to the responsibilities of other agents and norms correspond to conventions derived from the agent’s role in the environment. The goal created for the agent needs to be consistent and achievable [17]. According to the definition of the goal, the intentions for executions are planned. In the delegative model, the goals are defined as candidate goals and adopted goals [18]. Candidate goals are those that can be inconsistent internally while Adopted goals are the consistent and coherent ones in the BDI model. This model can even incorporate user-specified guidance and preferences from the user in the form of advice. The B-DOING framework lacks the distinctions between types of goals for proactive assistance, while the delegative BDI framework lacks the distinctions between types of motivational attitudes [16].

### 2.2. BDI Frameworks

Russel et al. [19] developed the agent factory framework as an open-source collection of various tools, platforms and languages that ultimately facilitate the development and development of multi-agent systems. Winikoff [20] built a highly portable, robust and cross-platform environment called JACK for building, running and integrating commercial-grade multi-agent systems. In the BDI framework called JADE [21], the agent platform can be distributed among different independent machines and controlled remotely. The configuration can even be changed at run-time by the moving agents from one machine to another one during the implementation. Braubach and Pokahr [22] developed a framework called *JADEX*, based on XML and Java, that follows BDI model and facilitates easier intelligent agent system construction with an engineering perspective. JASON is a super flexible platform developed as an extension of AgentSpeak [23] by [24], that implements the semantics of the language and provides a good platform for development of multi-agent systems with many customizable features. The comparison between different behavior model platform is given in Table 1.

ROS supports the C++ and Python programming languages for communication between different distributed nodes in its ecosystem. Because of various BDI frameworks available in Python, a pythonic framework is considered in this study.

### 2.3. Application of BDI Models in Robots

The behavior model was adapted to study the natural engagement of robots with humans to show exhibit proactive behaviors. The proactive behaviors in robots were imagined to increase the human-robot interaction and utility value in the use of robots. As such, the works related to proactive behavior in robots were initiated with mixed-initiative approaches. Finzi and Orlandini [25] developed an architecture based on a planner mixed-initiative approach for robots used in search and rescue operations. The study had a model-based execution monitoring and reactive planner for the execution of the tasks. Adams et al. [26] proposed an effect based mixed-initiative interaction approach for human-robot interaction. The robot took initiatives upon changes in human emotions like detecting drowsiness and inattentiveness. The robot, as developed by Acosta et al. [27] showed some proactive behaviors by monitoring activities and defining tasks as a schedule. Satake et al. [28] proposed a behavior model to initiate a conversation with pedestrians walking on the streets. The appropriate instant of time to start the conversation or interaction with people was studied in work done by Shi et al. [29]. Moreover, Garrel et al. [30] proposed a behavior model for a proactive model that tries to convince people to initiate a conversation with different behaviors and emotions. The study carried out by Araiza-Illan et al. [31] proposed the use of the BDI model to increase the level of realism and human-like simulation of the robots. An automated testbench was implemented for simulation of cooperative task assembly between a humanoid robot and people in the robot operating system and Gazebo. A soccer-playing robot based on BDI architecture was developed by Gottifredi et al. [32] which allowed the specification of declarative goal-driven behavior based on high-level reasoning and reactivity when required. The work of Duffy et al. [33] developed a multi-layered BDI architecture with an egocentric robot control strategy to make robots capable of explicit social behavior. Pereira et al. [34] proposed an extension to BDI architecture to support artificial emotions in the form of emotional-BDI architecture.

Given the current state of proactivity in social robots, this study tries to extend the capabilities of such robots to include vision-based activity in a social robot. The integration is based on a modular architecture onto which other logical blocks can easily be integrated for more advanced proactive behaviors in a think-like-human fashion.

## 3. Case Study Setup

In this section, we explain the use case scenario created to develop a proactive behavior in a social robot *Waldo*. Furthermore, we define a system framework based on existing technologies to integrate modular blocks of OpenCV and BDI reasoning with the ROS ecosystem with a detailed explanation of each step as follows.

### 3.1. Use Case Scenario

For this study, we consider a visual image-based activity for adding a proactivity behavior in the social robot. The indoor robot Waldo has cameras installed in its eyes which gather the image feeds from the environment. This image-feeds help to set up a belief about the situation. The camera feeds are processed using a module in OpenCV for person detection. This module establishes a belief for the robot concerning the presence of a person in the environment. In case a person detected by the OpenCV module, Waldo sets up a goal of greeting the person without any explicit commands from the person. In this experiment, the robot can perform two precise actions. Upon the detection of the person, using the BDI framework, the robot greets the detected people with a sentence. Upon continuous detection of the person for a fixed time, the robot changes its belief and offers additional help to the person by speaking a different sentence. For this work, the actions which Waldo can perform is only limited to speech, but advanced services can easily replace these actions. This provision is made in the experiment to show how beliefs can be changed according to the environment so that the actions taken by the robot can be relevant and more interactive.

### 3.2. Social Indoor Robot—Waldo

**Figure 1:**
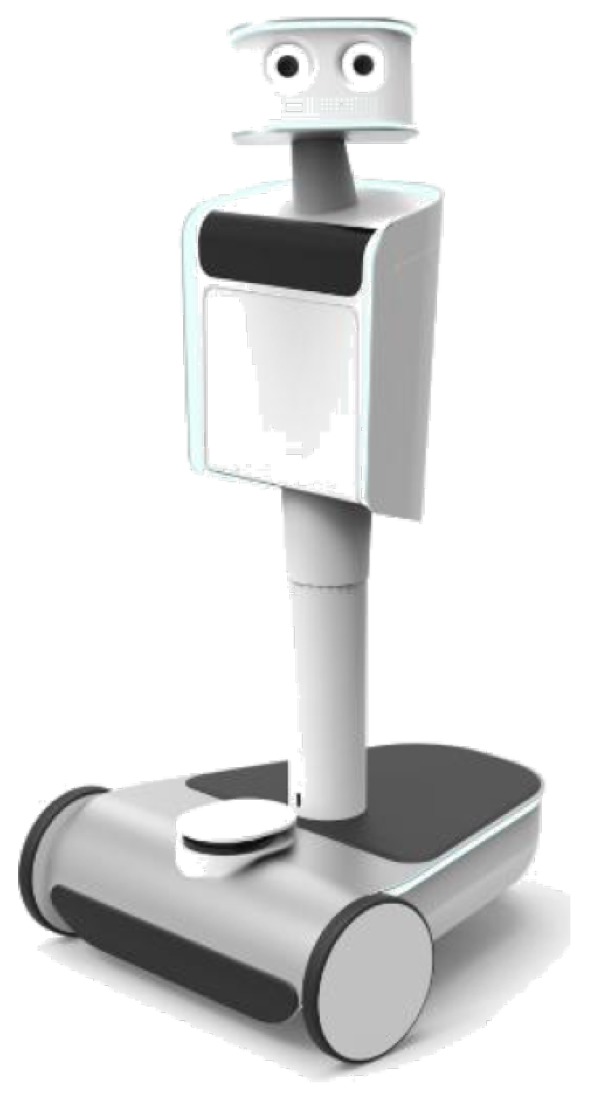
Waldo.

The robot under consideration for this study is Waldo, which is a multi-service robot manufactured by Immersive Robotics [35]. Waldo is a telepresence service robot with advanced vision capabilities. The robot has an Arduino card for basic control functions and a Linux card with ROS installed as an operating system for more advanced and elaborate functionalities. Waldo has an adjustable height of 130 cm to 170 cm. The robot is autonomous with LIDARs, sonars, microphones, and cameras. Waldo is built in a humanoid shape as an indoor social robot for a welcoming, talking, understanding and communicating with people. The movement of Waldo can be controlled remotely by using a mouse, keyboard, joysticks, pads, smartphones, tablets or any other desired peripherals. Waldo is shown in Figure 1.

### 3.3. System Overview

**Figure 2:**
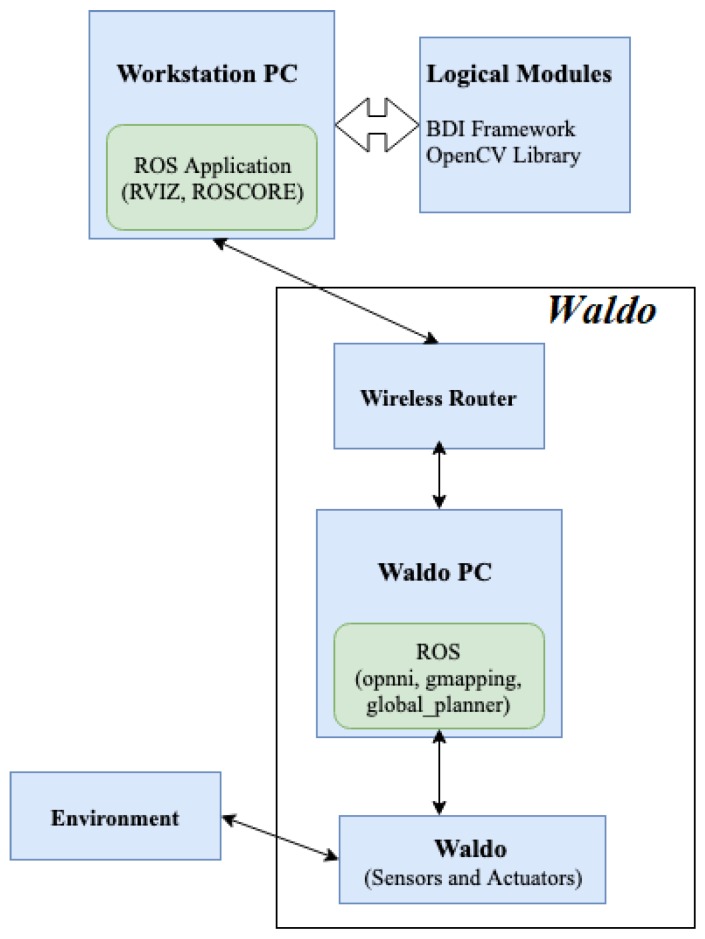
System overview.

The main goal of this study is to develop a flexible and modular framework using existing technologies that could facilitate the integration of different blocks as modules to the framework, which ultimately contribute in the development of proactive behaviors in a social robot controlled by ROS. The overall system overview for achieving the behavior model of the environment in indoor models is reflected in Figure 2. Different sensors installed in the indoor robot Waldo collect the information about the environment. The cameras, LiDAR and Kinect sensor help to collect information about the environment for a given instant of time. The information is relayed to Waldo PC, which is connected to the monitoring PC through a wireless connection. Because of the limited capabilities of Waldo PC, the compute-intensive logical modules can be run on a more powerful monitoring PC. The logical models are responsible for deriving various knowledge from the data about the environment collected by sensors of the robot. Based on this knowledge base, the BDI framework establishes the beliefs and set up goals for any given instant of time. The framework also chooses a set of actions from the predefined plan list to accomplish the goal. The actions are relayed to *Waldo* PC through a wireless connection that directs different actuators in *Waldo* to perform a wide range of action. The internal mechanisms in ROS manage the communication between the nodes and *Waldo* PC. The Waldo PC, which is equipped with Linux and ROS, can have several logical modules installed on it or monitoring PC to establish one or more beliefs about the environment. The modules in the logical layer can be BDI framework, OpenCV for vision processing and other intelligent bricks for establishing important beliefs about the environment. The BDI framework responds to the established beliefs and set up goals for any instant of time. The engine in the framework chooses a plan of actions from the predefined libraries for goal accomplishment. The plan execution is relayed to Waldo PC, which generates actual actions in the robot to respond to the environment. The flow operation can be monitored using a monitoring PC connected to Waldo PC over a wireless network.

### 3.4. BDI Modeling

The BDI modeling of the problem should be able to answer the following question effectively and efficiently.

- When to act? The robot works whenever it detects a person inside the room. So, an efficient mechanism for the detection of a person should be integrated into the robot. An effective block to trigger actions has to be adopted.
- What actions to take? The actions which are expected to be made by robots hugely depend upon the detection of the people. The action can be a simple greeting message delivered to the person or message delivered to the person offering some help or no action at all.
- How to perform the actions? Based on the set of beliefs about the environment, the robot can decide to take action. For any case of detection of a person, the robot employs its text to speech node for speaking out sentences to either greet or offer help to the people. For non-detection of the person, the robot can deliberate itself to stay idle or go to sleep mode.

The knowledge about the surrounding is collected by the cameras installed on the eyes of the robot. For the experiment, the camera feeds are only used. The person detection block from OpenCV is used to define the belief for the system design explicitly. Moreover, the goals and actions are defined accordingly to realize a use case of proactive behavior. The possible actions which the robot could take were limited only to the speech. The set of beliefs, desires, and intentions as per BDI model for the case study are defined as follows:

**Belief**: personDetected(“Yes”), personDetected(“No”) and personDetected(“Next”)

**Desire**: DoNothing(), GreetPeople() and OfferHelp()

**Intentions**: stayIdle(), speak()

The PROFETA framework can be implemented for behavior modeling using the following steps:

**Algorithm 1** Implementation of PROFETA framework.1: Import necessary PROFETA libraries2: Define beliefs and goals as classes in the script3: Define user actions by creating classes and overriding the method execute()4: Start PROFETA engine5: Define rules by using declarative syntax6: Run the engine

Moreover, PROFETA framework also facilitates the definition of sensor class which can itself add or remove a set of beliefs depending upon the environment. This can be done in PROFETA by declaring a subclass Sensor, overriding the sense() method and informing the PROFETA engine about the addition of a new sensor in the program. PROFETA uses declarative language so as to express the behaviors of agents. The declarative syntax for the behavior of an agent is described below:

“Event”/“Condition” >> “setofActions”

In this declarative syntax, an event can be any one of belief assert or retract, goal accomplishment or request or even goal failures. The condition in the syntax refers to a particular set of knowledge base while actions can be goal accomplishment request, user-defined set of actions, or adding or removing beliefs. This syntax can be exemplified as:

+objectAt(“A”, “B”)/objectGot(“no”) >> [moveTo(“A”,“B”), pickObject()]

### 3.5. Person Detection Using OpenCV

**Figure 3:**
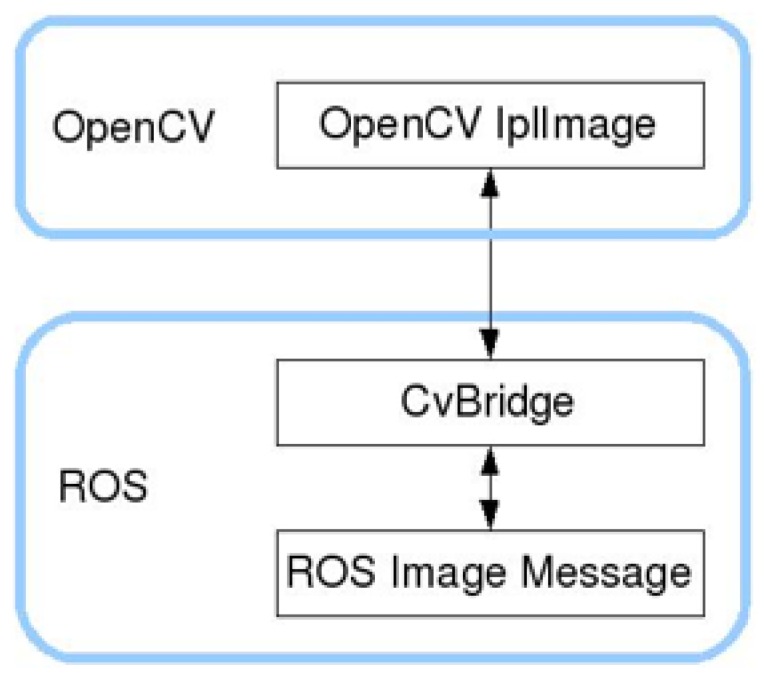
CvBridge interface.

For person detection, open-source computer vision library (OpenCV), a freely available open-source library for computer vision and machine learning, is used. The libraries and algorithms in OpenCV are directly used in the experiments to detect a person based on the histogram of oriented gradient (HOG) features and support vector machine (SVM) classifier. The performance improvement of the OpenCV algorithms for person detection is beyond the scope of this work. ROS has its own image format used for communication between nodes through subscription and publishing. This image format has to be converted into OpenCV format to use the OpenCV libraries for person detection. CvBridge, a library in ROS, facilitates the conversion of ROS images to OpenCV image format and vice-versa. The CvBridge interface is represented in Figure 3.

### 3.6. Experimental Setup

The test use case is implemented in Waldo with the works distributed over two PCS. The workstation PC has Ubuntu 16.04 installed on an Intel i5-5300U processor with ROS Kinetic whereas the Waldo PC has the same operating system installed on an Intel(R) Atom(TM) processor. The proactive behavior of *Waldo* is well implemented using the BDI framework of PROFETA.

## 4. Results and Discussion

In this section, we present the validation of the proposed system design for the integration of various logical modules like the BDI model and OpenCV into ROS for proactive behaviors. The capabilities of the proposed framework are validated using a qualitative approach, where several features such as modularity, flexibility, and rational work distribution are investigated. Furthermore, we explain the associated findings in detail and include a quantitative analysis of the results for logical OpenCV module.

### 4.1. Work Distribution in Proposed System Architecture

One of the key features expected from the proposed framework is the rational distribution of works required in the development of proactive behaviors in the robot. The study implements the test use case in ROS with an overlaid layer of the BDI framework of PROFETA. A module in the OpenCV library does the image processing. The proposed system design distributed the operations over two PCs, Waldo PC, installed in the robot and Workstation PC. The test experiments utilized the distributed working architecture of ROS as the design offloaded the Waldo PC from heavy processing of image feeds collected from the cameras. The bulky and more compute-intensive modules of person detection and practical reasoning (BDI framework) are installed in a comparatively powerful Workstation PC. The actual actions and the management of different ROS nodes are handled in Waldo PC. These actions are not compute-intensive. As such, the proposed architecture supports a reasonable work distribution in the development of proactive behaviors. Consequently, more advanced activities can be thought of as an extension as more compute-intensive modular blocks can be easily integrated into the robot, thanks to the architecture. More powerful machines can assume the role of Workstation PC for such capabilities while Waldo PC can assume the light roles of information collection and effectors. Moreover, the framework allows us to add additional computational devices to consider different workloads required for various proactive behaviors. Such capabilities make the proposed framework flexible as well.

### 4.2. Validation of Proposed System Design with Test Use Case

**Figure 4:**
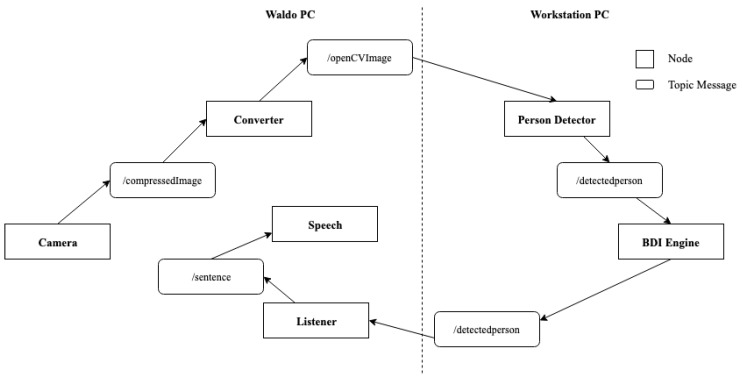
Interaction between different nodes in robot operating system (ROS) ecosystem.

The validation of the proposed system design focused on investigating whether the features of flexibility, modularity, and rational work distribution are achieved during the exhibition of proactive behavior by Waldo. For validating the proposed system design, we created several distributed nodes within and outside the ROS ecosystem. There is one ROS node each for camera feed, person detection block and speech block. The different ROS nodes communicate with each other through the topic messages. Roscore manages the communication between the nodes. As required, the nodes can be created and removed for adding or removing functionalities. The nodes can be created in any of the computing devices available within the proposed framework, providing flexibility and modularity. Initially, the camera node publishes the image feed collected by the eyes of Waldo. The converter node, with CvBridge interface, subscribes to the topic messages of camera node and converts the ROS images to OpenCV images. The node then publishes the converted images. There is a node called person detector running in Workstation PC, that subscribes to the converted image messages. This node executes the person detection module of OpenCV library. There is an additional node called BDI engine in Workstation PC that subscribes to the messages about person detection published by the person detector. BDI engine executes all the necessary behavior modeling to publish the action to be done by the robot finally. There is another node, listener, that subscribes to BDI engine node and publishes messages for actuators in the robot to undertake the actions. The speech node that subscribes to listener makes the robot speak out the sentences to achieve the goal. The entire interaction between different nodes in a widely distributed ROS ecosystem is shown in Figure 4. As can be seen in the figure, different workloads required for the proactive behaviors are distributed over Waldo PC and Workstation PC. Such rational work distribution is one of the strengths of the proposed framework, where computing devices can be easily added or removed based on the requirements. Furthermore, additional logical blocks/modular can be created as new nodes within the ROS ecosystem in the proposed framework to develop additional functionality in the social robot. In our study, we created a node for the movement of the head of the robot during the person detection to demonstrate the modularity in the proposed framework. The newly created node communicates with the person detector node to create any movement.

### 4.3. Performance Analysis

For the performance analysis and evaluation, Waldo is kept at a fixed position in the corridor. The eyes of the robot, which have cameras, are at the height of about 162 cm. The tests are carried repeatedly where both natural and artificial lights influence the lighting condition.

**Figure 5:**
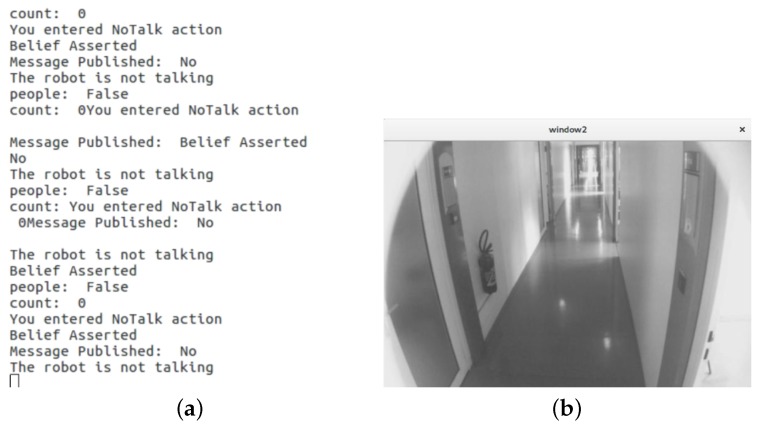
Case: no person detected (**a**) BDI execution and (**b**) image feed collected by eyes of Waldo.

**Figure 6:**
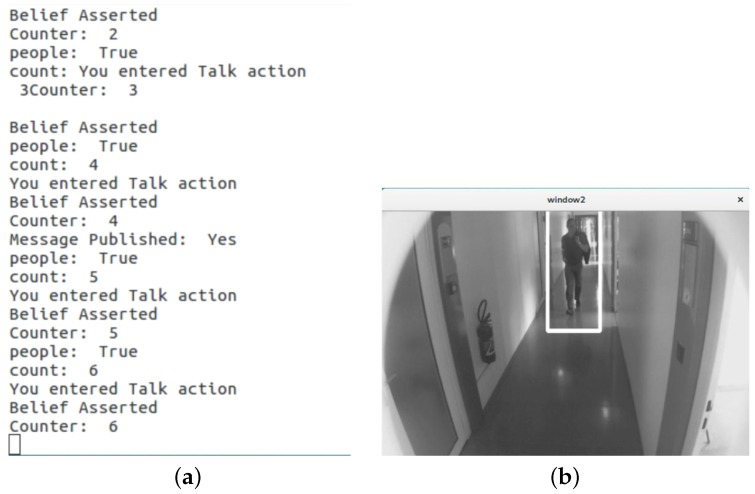
Case: person detected (**a**) BDI execution and (**b**) image feed collected by eyes of Waldo.

Figure 5 and Figure 6 represent the execution of BDI actions and image feeds collected by the camera installed in the robot, for no person detected and person detected cases, respectively. When a person is not detected in the image feed, the BDI engine establishes the belief about the environment and create a goal of not greeting the people. Accordingly, the engine selects the action of NoTalk to achieve the goal, as shown in Figure 5a. Similarly, when a person is detected, the BDI engine asserts a belief of encountering a person, enabled by the person detection module running on the Workstation PC. Based on this belief set, the engine has to set up a goal of either greeting the people or offering additional help to the people. For the distinction between the two, we add logical operation of tracking for how long the person has been encountered, as represented by the counter in Figure 6a. Based on the value of counter and person detected in the image feed, the BDI engine establishes two distinct belief sets requiring two different goals. The greeting action to achieve the goal of greeting people is represented as talk action in Figure 6a. The help action is executed to achieve the purpose of offering help to people when the person is detected continuously for a more extended period.

Moreover, we tested the working of the overall system considering different distances from the robot in two different scenes. The analysis of the performance is presented in Table 2 and Table 3.

**Figure 7:**
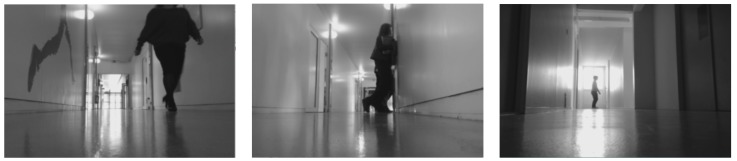
False negatives.

**Figure 8:**
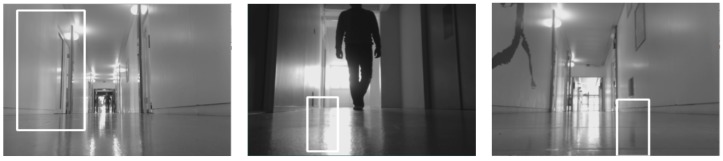
False positives.

**Figure 9:**
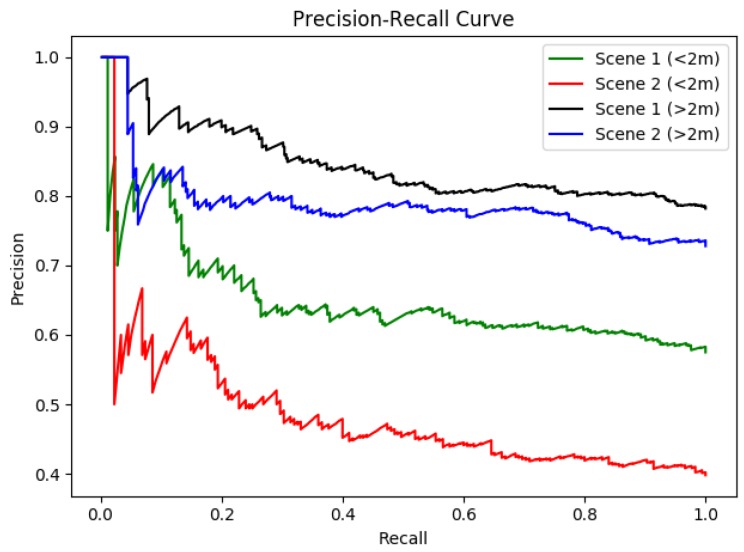
Precision-recall curve.

The performance analysis of the entire system showed that the robot performed quite poorly for the region beyond 2–10 m. The respective values of precision and recall for Scene 1 are 0.822 and 0.782, while the same for Scene 2 are 0.756 and 0.728. Scene 2 had more lighting inconsistencies when compared to Scene 1. The false negatives and positives (as shown in Figure 7 and Figure 8) during the test were caused by the change in lighting conditions brought about by the movement of objects. Moreover, such negatives were caused by the shadows of the people formed by several sources of lights (natural and artificial). Because of the unequal distribution of data sets, we also calculated the F1-Score for each scenario. The best F1-score was obtained for Scene 1 as 0.801 for the region of 2–10 m. The high value of F1-score (closer to 1) shows the efficiency of the module when detecting the person so that the robot can exhibit proactive behavior in greeting and offering help to the person detected. Similar to the analysis given by precision and recall, F1-score for Scene 2 in the region of <2 m is the lowest, highlighting the fact that the logical block does not perform so effectively in that location. Furthermore, we plotted the precision-recall curve for our experiments, which are shown in Figure 9. The curve also confirms the findings and establishes the best performance of the module in Scene 1 (2–10 m region) as the area under the curve for the case is the highest (see Figure 9). The performance of the module is affected by several factors such as artificial light, shadow, multiple sources of light and the distance between the camera and the person. The accuracy of the system design in the given test case can be improved by enhancing the performance of the OpenCV logical block.

### 4.4. Limitation

For this study, the image frames without the entire body of the person are not considered. Since the cameras are placed at a certain height and the angular movement of the head is limited, the camera cannot cover the distance that was less than a meter. Thus, the region in front of the robot, less than 2 m away, is a blind spot. Such blind spots give poor performance in relation to person detection. Moreover, beyond the distance of about 10 m, the person detection module is not able to detect the person. As for the system design, the architecture still offers modularity and flexibility. The accuracy of the entire system is largely dependent upon the logical block used to develop proactive behaviors in the robot. Apart from the limitation in the logical block, the study focuses more on the qualitative validation of the proposed framework, where the features, such as modularity, flexibility, and rational work distributions, are investigated. The work is primarily determined in developing a basic flexible foundation using existing and freely available tools for the development of proactive behaviors in social robots. Because of no readily available comparable architecture and case studies, a comparative analysis is not included in the paper. Moreover, the advanced capabilities of the social robot are also not considered as of now. These limitations of the study will be enhanced in the future.

## 5. Conclusions

The recent rise of humanoid and animaloid indoor social robots in commercial spaces, including home has necessitated the “act-like-human” behavior in those robots for a more friendly human-robot interaction. The proactivity in such social robots adds more utility to the robots. As such, in this paper, we presented a validated use-case of such proactive behavior in an indoor commercial social robot, *Waldo* enabled by the behavior model framework, PROFETA. We clearly defined a fundamental system architecture with the features of flexibility, modularity, and rational work distribution to integrate the BDI framework into the distributed ecosystem of ROS. In the architecture, multiple ROS nodes can be independently created over multiple machines, connected by wireless communication. We demonstrated how an external module, such as OpenCV library, can be used to enhance the capability of an indoor robot in a plug-and-play fashion. We expect the proposed system architecture to lay a solid foundation to develop a wide range of proactive behaviors in an indoor social robot to behave and act like a human. Such behaviors can be possible with the addition of various logical modules into the proposed architecture.

This work validated the working of the proposed architecture with the basic actions of the robot. Preliminary works are underway to integrate the blocks of artificial intelligence into the proposed architecture to develop more intelligent actions in the robot. The expansion of the actions which Waldo can perform, is also ongoing. The future work can be centered around establishing a more accurate belief within the human reasoning paradigm, BDI, by using data collected by multiple sensors in the robot. Other than the camera, LiDAR and Kinect sensor can be used to represent the state of the environment better. In terms of the action of the robot, further works can focus on integrating the autonomous navigation of the robot to achieve the goal set up based on different beliefs. The addition of learning mechanisms, to keep improving, can be thought of as an essential extension.

## Figures and Tables

**Table 1:** Comparison between different beleif–desire–intention (BDI) platforms.

Name	Primary Domain	Open Source	Learn-Ability	Programming Language
Agent Factory	General purpose agent based	Yes	Average	Java, AgentSpeak
JACK	Dynamic and Complex environment	No	Easy	Java
JADE	Distributed applications composed autonomous entities	Yes	Easy	Java
JADEX	Distributed applications composed autonomous BDI entities	Yes	Easy	Java
BDI4JADE	Enterprise application	Yes	Average	Java
JASON	Distributed applications composed autonomous BDI entities	Yes	Easy	Java
PROFETA	Distributed applications composed autonomous BDI entities with AI applications	Yes	Easy	Python
SPYSE	Distributed AI applications	Yes	Average	Python
SPADE	Distributed Multi-agent	No	Average	Python

**Table 2:** Distance consideration for the validation of the work.

Distance	Scene 1			Scene 2		
**(of Person from Camera)**	**True Positives**	**False Positives**	**False Negatives**	**True Positives**	**False Positives**	**False Negatives**
<2 m	253	160	187	175	160	265
2–10 m	401	87	112	354	114	132

**Table 3:** Precision and recall evaluation.

Distance	Scene 1			Scene 2		
**(of Person from Camera)**	**Precision**	**Recall**	**F1 Score**	**Precision**	**Recall**	**F1 Score**
<2 m	0.612	0.575	0.593	0.52	0.398	0.451
2–10 m	0.822	0.782	0.801	0.756	0.728	0.742

## Appendix A

**Table A1:** Reactive vs. Proactive Behaviors.

	Reactive	Proactive
User	Could you help me?	
		**It seems like your computer**
Robot	Yes, What is wrong?	**is not responding, let me**
		**know if I can do anything?**
User	It seems like my computer	Could you help me?
	is not responding	
Robot	You should end the process	You should end the process
	by pressing ALT+CTRL+DEL.	by pressing ALT+CTRL+DEL.
User	Won’t that restart my computer?	Won’t that restart my computer?
Robot	Don’t worry; it will just	Don’t worry; it will just
	end the process.	end the process.
User	Hmm, it’s not doing anything.	OK
Robot	Press ALT+CTRL+DEL together.	Press ALT+CTRL+DEL together.
User	Ok and now?	
		Then you should end the process.
Robot	Then you should end the process.	**Do you have a back-up?**
User	But then I lost my work?	I’m not sure.
Robot	Don’t worry your computer makes a	Don’t worry your computer makes a
	backup every five minutes	backup every five minutes
User	How can I find a backup?	Where is my backup?
Robot	You can open your program	To find your program, you can
	again and select the file.	open your program again and
		select the file. Try it!

Note: The bold texts are the proactive behaviors (adopted from [13]).
